# Towards a Greater Contextualization of Music and the Arts in Aging and Cognitive Disorders

**DOI:** 10.1093/geroni/igaa057.3083

**Published:** 2020-12-16

**Authors:** Desmond O’Neill

## Abstract

There is an increasing prominence of arts and cultural interventions related to aging and cognitive disorders in the scholarly literature and at gerontological conferences. However, the mechanisms of the salience and relevance of aesthetics, culture and leisure in the lives of older people remains unclear. One aspect which has emerged is that of aesthetic deprivation and its consequences for well-being. This symposium aims to provide perspectives from a range of researchers involved in programs of research and implementation to try to contextualize and better understand the perspectives of older people, arts practitioners and therapists the place and context of arts, culture and leisure in optimal aging.

